# Comparing conventional and action video game training in visual perceptual learning

**DOI:** 10.1038/s41598-024-71987-y

**Published:** 2024-11-13

**Authors:** Maggie S. Yeh, Tan Li, Jinfeng Huang, Zili Liu

**Affiliations:** 1 Department of Psychology, University of California Los Angeles, Los Angeles, CA USA; 2 Department of Psychology, Hebei Normal University, Shijiazhuang, China

**Keywords:** Sensory processing, Perception, Human behaviour

## Abstract

Action video game (AVG) playing has been found to transfer to a variety of laboratory tasks in visual cognition. More recently, it has even been found to transfer to low-level visual "psychophysics tasks. This is unexpected since such low-level tasks have traditionally been found to be largely “immune” to transfer from another task, or even from the same task but a different stimulus attribute, e.g., motion direction. In this study, we set out to directly quantify transfer efficiency from AVG training to motion discrimination. Participants (*n* = 65) trained for 20 h on either a first-person active shooting video game, or a motion direction discrimination task with random dots. They were tested before, midway, and after training with the same motion task and an orientation discrimination task that had been shown to receive transfer from AVG training, but not from motion training. A subsequent control group (*n* = 18) was recruited to rule out any test–retest effect, by taking the same tests with the same time intervals, but without training. We found that improvement in motion discrimination performance was comparable between the AVG training and control groups, and less than the motion discrimination training group. We could not replicate the AVG transfer to orientation discrimination, but this was likely due to the fact that our participants were practically at chance for this task at all test points. Our study found no evidence, in either accuracy or reaction time, that AVG training transferred to motion discrimination. Overall, our results suggest that AVG training transferred little to lower-level visual skills, refining understanding of the mechanisms by which AVGs may affect vision.

*Protocol registration* The accepted stage 1 protocol for this study can be found on the Open Science Framework at https://osf.io/zdv9c/?view_only=5b3b0c161dad448d9d1d8b14ce91ab11.

The stage 1 protocol for this Registered Report was accepted in principle on 01/12/22. The protocol, as accepted by the journal, can be found at: 10.17605/OSF.IO/ZDV9C

## Introduction

Visual learning is essential for interacting with and interpreting an individual’s environment. The ability to improve visual skills plays a role in various forms of skill acquisition in fields such as sports, education, and medicine. From a scientific perspective, visual learning is a mechanism that provides externally observable improved behaviors that can in turn be utilized to infer further understanding of the workings of the brain. For visual perception, therefore, it is advantageous to identify and understand potential training methods, such as video games.

What is formally known as visual perceptual learning (VPL) refers to improvement of visual perceptual skills after some visual training. There is extensive literature studying perceptual learning of fundamental visual dimensions such as orientation^1–3^ and motion direction^4,5^.

Traditional VPL research has found that perceptual training tends to result in specific learning. That is, the improvement observed after traditional VPL training is specific to stimulus attributes used in that training, but does not transfer to other values of a trained attribute^6–8^. For example, participants trained to identify the target grating from two vertically oriented gratings dropped to pretraining performance when the same gratings were rotated and presented horizontally^3^. These participants attained a level of mastery of the discrimination task, but only with the originally trained stimuli. The exact mechanism behind VPL remains under active debate in the VPL field, but this specificity is often interpreted to suggest that VPL is occurring at low-level of processing in the brain, e.g., in the primary visual cortex (V1), where neurons respond to specific visual characteristics. In other words, specificity would suggest that VPL is a kind of template learning, that is unable to generalize beyond the learned exemplars.

However, it is unlikely that VPL only occurs in V1. There are exceptions to the trend of specific learning in VPL, where studies have added manipulations to their training paradigm, resulting in transferable learning. One well-known study^9^ introduced the concept of “training plus exposure,” where training one feature at location 1 and exposure to an irrelevant second feature at location 2 resulted in transfer of learning of the first feature to the second location (but see contrasting evidence^10–12^). In other words, training plus exposure was able to overcome location specificity (see other examples^13,14^). Other earlier studies have also explored factors that may influence transfer, such as the role of task difficulty^15–18^. These studies have led to various theories regarding the mechanisms of VPL involving factors such as when activation of different visual areas occurs, or how connections between different levels of visual processing might be modified^19–23^. Other researchers have instead focused on the functional mechanisms of visual learning rather than the physical mechanisms, using such models as signal-detection theory^13^.

In comparison, studies of the effect of action video games (AVGs) on visual learning have commonly reported generalization of AVG-induced learning to untrained stimuli^24–26^, although there are some notable studies that have failed to replicate AVG-induced learning^27,28^ and articles that have pointed out methodological issues with AVG learning results^29–31^. AVG training is a paradigm where participants play action video games and are tested for improvement in visual skills. The games used in this training typically are first-person shooter (FPS) games, where the player views the screen from a first-person perspective, and must identify and shoot various targets that possess their own unique movements and behaviors. For example, one early study trained participants with an FPS game and found generalization to flanker compatibility, enumeration, and attentional blink tasks^32^. These tasks used stimuli that differed significantly from the video game graphics that participants trained on, suggesting that AVG training generalized to a wider range of visual stimuli. Furthermore, the tasks did not precisely replicate any sub-tasks that made up the gameplay of the FPS game, suggesting that AVG training generalizes to a variety of visual skills. The generalizability of AVG suggests that it induces some form of general rule learning, regardless of whether participants were aware of such rules.

Despite the promising extent of transfer reported in AVG studies, the vast majority of AVG research has focused on attention-based visual skills such as visual search^33,34^ and visual working memory^25,35,36^. In contrast, VPL typically focuses on learning basic dimensions of visual features, such as orientation and motion direction. Overall, only a few studies have examined whether generalizability of AVG extends to the stimuli and tasks typically used in VPL research. These include a study that assessed video game-induced improvement in acuity, spatial and temporal contrast sensitivity, contrast threshold in noise, global motion perception, and useful field of view^37^; another that tested the effect of video game training on the contrast sensitivity function^38^; and a third that tested on an orientation discrimination task with variable external noise^39^. Of these studies, several have reported evidence of positive benefits of AVG training for lower-level visual skills^38–40^, but the overall number of such studies remains low (< 10). That is, there are still, relatively speaking, few studies testing the effects of AVG on visual skills utilizing low-level fundamental visual characteristics, and we would like to verify and extend the results of those studies that have done so^38–40^. There is also the fact that the results of existing AVG learning studies remain controversial (e.g.^31^), and so we wish to examine the claim of AVG transfer to vision using more rigorous traditional methodology from VPL literature.

AVG may induce some form of rule learning that traditional VPL training does not do. As a possible explanation for this difference between AVG and traditional VPL training, we hypothesize that AVG is a form of varied training. Varied training involves varying the stimuli or tasks used for training^41^. The reported effects of varied training include better performance on a novel stimulus that was actually used for training the comparison group – the constant training group – than the performance of that comparison group. This suggests that varied training is beneficial for generalization of learning. The reason for the effectiveness of varied training may be that the variety of stimuli presented in varied training enables learning of a broader and more abstract concept, rather than focusing on the specific attributes that might be more useful in a narrower training. For example, traditional VPL training may train participants to discriminate between 43 and 47 degrees in directions. Whereas in AVG, a range of stimuli such as 43 and 47 degrees, 83 and 87 degrees, or 133 and 137 degrees may be also presented in training, such that discriminating two directions that differ by 4 degrees may be better learned no matter what the average of the two directions is^5,42^. A participant trained with AVG may therefore be better able to learn underlying rules of direction discrimination from such variety of stimuli, while a traditional VPL-trained participant may struggle to learn such a rule due to the lack of various examples provided.

The varied training hypothesis may also potentially explain some of the VPL studies that found transfer of learning. One suggested explanation for how methods such as “training plus exposure” may encourage transfer is that the secondary training location serves as a prime for that location^9^. In other words, exposure to a higher variety of locations allows for transfer of learning to multiple locations. Varied training may serve as an expansion of that priming effect, where the trainee is exposed to a wide variety of locations and features, thus allowing for generalized visual learning. This had been termed “rooting” in an earlier study^13^.

In order to better understand AVG’s generalized learning, we directly compared AVG with traditional psychophysics VPL training. This comparison could provide not only insight into the differences between the two training methods, but also additional evidence of whether AVG can generalize beyond attention-based tasks. In addition, the proposed hypothesis of varied training would also suggest that the characteristics of AVG-induced learning may differ from the characteristics of traditional VPL.

One way of comparing AVG and VPL training is to compare their rates of learning. Our reasoning was that AVG may demonstrate a slower rate of learning, because learning an abstract rule from a more complex video game may take longer than learning a specific instance from a more concrete VPL training task. There is also evidence that suggested that varied training required more training in order to reach the same criterion of learning comparable to constant training^43^. The learning rates of AVG and traditional VPL training have not previously been compared, but doing so would provide evidence of potential mechanistic similarities and differences between the two methods.

In the AVG literature, one review suggested that 20 hours (h) of training was the minimum required in order to observe significant learning^44^, although in the literature there was a range of training times from 10 to 50 hours^32,44–47^. In contrast, VPL literature typically does not quantify training in terms of hours, but rather in terms of number of sessions completed, and research on minimum training required is scarce. One study suggested that as few as five trials per condition per day was sufficient to obtain significant learning on a texture discrimination task^48^. Another study suggested that a minimum of 400 trials per session was required for learning a Chevron discrimination task^49^. However, it is not unusual to see VPL studies that required participants to complete weeks of training sessions^11,50^.

Using equal training time, we tested our hypotheses by having participants train either on AVG or psychophysics tasks. This would allow us to assess whether the amount of transfer to the psychophysics tasks from AVG training is comparable to that from traditional psychophysics training of the same task (we already expected minimal transfer from the trained psychophysics task to the untrained psychophysics task).

All participants completed a pre-test and post-test consisting of two psychophysics tasks to assess performance before and after training. The psychophysics task used for training was identical to one of the tasks used in pre- and post-tests. This was the matched task, while the other task used in testing served as a nonmatched task. This allowed us to test whether the AVG training was able to induce learning in the two psychophysics tasks, and if this transfer was more than that from the matched to the nonmatched psychophysics task. There was also the question of potential differences in rate of transfer. Therefore, participants also completed a middle test after 9.75 h of training, in addition to the pre- and post-training tests, with 19.5 h training total.

In sum, we planned to test four hypotheses. First, after completing the entirety of training, we predicted that those trained with AVG would demonstrate comparable levels of learning (in the form of improved test performance) on a matched psychophysics test as compared to participants trained on a psychophysics task.

Second, for the nonmatched psychophysics test, we predicted that participants trained with AVG would demonstrate more learning post-training than the participants trained with psychophysics. In other words, the AVG participants would show transfer from AVG to both psychophysics tasks after completing all training. This would take the form of an interaction between training type and time, comparing pre-test to post-test.

Third, for the matched test, we predicted that those trained with AVG would demonstrate an overall slower rate of learning than the psychophysics training group. This would take the form of two interactions. The first interaction is between training type and time, comparing learning from pre-test to mid-test (9.75 h into training). We expected the psychophysics training group to show more transfer than the AVG group at this point. The second interaction is similar to the first but compares learning from mid-test to post-test. We expected for AVG to show more transfer than the psychophysics training group—this would indicate slowing down or even a plateau for the psychophysics training group after the first half of training while the AVG group continued to improve.

Fourth, for the nonmatched test, we predicted that those trained with AVG would show more transfer than those trained with psychophysics. This would take the form of at least one main effect and one interaction, from pre-test to post-test. The main effect would be of time, and the interaction would be between training group and time. This is because AVG was predicted to transfer, whereas the motion training was predicted not to transfer to the orientation task. However, we were uncertain whether these effects would occur already at mid-test, or not till post-test. Table 1 summarizes the hypotheses and hypothesis testing plan.

**Table 1:** Design table.

Question	Hypothesis	Sampling plan (e.g. power analysis)	Analysis plan	Interpretation given to different outcomes
Does action video game training cause visual perceptual learning comparable to traditional psychophysics training, after sufficient training?.	Participants will show comparable improvement on the matched psychophysics test after full training is completed, regardless of training condition.	We propose that n = 64 participants will be sufficient for our study based on our *a priori* power calculations.	We will conduct a repeated-measures 2 × 2 ANOVA examining the effect of training type and time on performance for each psychophysics test. If we obtain results with p > .05, We will follow up with equivalence testing to determine whether the mean percentage improvement (MPI) observed in the AVG condition is statistically equivalent to the MPI observed in the psychophysics training condition.	If there is no significant main effect for training type for the psychophysics test matched to the training task, this will be interpreted to mean we can not reject our hypothesis—that action video game training causes comparable improvement to traditional psychophysics training. Equivalence testing will then be used to assess whether we can conclude equivalence of the two groups, which would support our hypothesis. If there is a significant effect of training type, such that psychophysics training causes larger improvement, this will be interpreted to mean that action video game training may not be as effective as psychophysics training for improving visual perceptual skills.
Does action video game training cause learning that generalizes to a variety of visual perceptual skills?.	Participants trained on the action video game will show significant improvement on all psychophysics tests after training.	We propose that n = 64 participants will be sufficient for our study based on our *a priori* power calculations.	We will conduct a repeated-measures ANOVA examining the effect of training type and time on performance for each psychophysics test. We will then conduct t-tests to further examine any significant results.	If there is a significant main effect of time for participants trained on the action video game for all tests, this will be interpreted to support our hypothesis. That is, that action video game training causes significant improvement on a variety of visual perceptual skills. If there is no significant main effect of time for any of the tests, then this will be interpreted to mean that AVG training has no effect.
Does action video game training cause more generalizable visual perceptual learning at the cost of slower learning rate?.	Participants trained on the action video game will show significant improvement on all psychophysics tests, but only after all training has been completed. In contrast, participants trained on the psychophysics task will show significant improvement on the matched test after a shorter period of training, but will show smaller increases in performance after further training.	We propose that n = 64 participants will be sufficient for our study based on our *a priori* power calculations.	We will conduct a repeated-measures 2 × 2 ANOVA examining the effect of training type and time on performance for each psychophysics test. There will be one ANOVA for each possible combination of time points. If any of our ANOVAs produces a result with p > .05, we will follow up with equivalence testing to determine whether the MPI observed in the AVG condition is statistically equivalent to the MPI observed in the psychophysics training condition.	For the matched test, if there is a significant interaction for the ANOVA comparing pre- to mid-test, this supports part of our hypothesis, as it suggests that psychophysics training produces a faster rate of learning on a visual task over a short period. If this is paired with an interaction from the ANOVA comparing mid- to post-test, this supports the other part of our hypothesis, as it suggests that AVG continues to produce learning in the second half of training, while psychophysics training may not improve much over a longer period. Finally, if there is only a significant main effect of time in our ANOVA comparing pre- to post-test, then this fully supports our hypothesis, as it suggests that the overall improvement between both training methods is similar. Therefore, two of our ANOVAs would have to produce an interaction to support our hypotheses about learning rates, while our third ANOVA would need to produce only a main effect in order to support our hypothesis about overall comparable improvement. For our nonmatched test, if there is only a main effect of time for the ANOVA comparing pre- to mid-test, this will support our hypothesis that neither AVG nor nonmatched psychophysics training will cause significant improvement from a short period of training. If there is a significant interaction for the ANOVA comparing mid- to post-test, this will support the other part of our hypothesis, as this would suggest that AVG is able to cause more improvement than psychophysics training on a nonmatched test. Finally, if there is a significant interaction from the ANOVA comparing pre- to post-test, then this fully supports our hypothesis, as this would suggest that AVG is able to transfer to multiple visual tests, while psychophysics training is only able to transfer to the matched test. For any of these ANOVAs, a lack of significant interaction suggests that AVG and psychophysics training may transfer comparably where they were predicted to differ. In contrast, the presence of a significant interaction where one was not predicted suggests that AVG and psychophysics training differed in transfer where they were predicted to be comparable. One such alternate explanation is that action video game training may simply not be effective for training visual perceptual skills, which may cause a simple main effect of training type.

## Methods

### Ethics information

Our research complied with all relevant ethical regulations. Our study protocol was approved by Hebei Normal University (Protocol ID: 2020LLSC050) and the University of California, Los Angeles (UCLA) General IRB (Protocol ID: 21–000,928). Informed consent was obtained from all participants. Participants were compensated either monetarily (Hebei Normal University), or with course credit (UCLA). Compensation occurred when participants completed their final experimental session, or at voluntary early termination of participation, whichever occurred earlier.

### Design

#### Survey

All participants completed a survey prior to the experiment. The survey included demographic questions regarding gender and age. Participants were also asked about motion sickness, normal vision, and prior experience with psychophysics tasks. The bulk of the rest of the survey consisted of questions regarding the participant’s video game experience in the past year, and asked participants for estimates of time spent playing games and examples of commonly played games. Finally, we screened for participants who had neither significant experience in video games nor in psychophysics tasks as our experimental participants. Additional survey details can be found in the Supplementary information.

#### Experimental design

This study consisted of a 3 × 3 design, containing one between-subjects variable, and one within-subjects variable. For the between-subjects variable, participants were assigned to one of three training conditions: AVG, psychophysics training, or a no-contact control. Note that Table 1 mentions two training conditions, but the final experimental design included an additional control group. For the AVG and psychophysics conditions, note that we separated participants by gender and randomly assigned them to the two training conditions within each gender group such that the gender ratio of the two training groups was as similar as possible. This is because prior video game studies have often found that recruiting participants who have no prior video game experience tends to result in primarily female participants. Whether this is due to cultural factors or self-selection resulting from perceptual differences is unclear, and so we attempted to ensure that there was the same gender ratio in all training conditions.

There was also one within-subjects condition. Namely, the duration of training before a given test: 0, 9.75, or 19.5 h. There were two measures at each time point: an orientation discrimination task and a motion direction discrimination task. All participants took a test consisting of these two psychophysics tasks at 0th, 9.75th, and 19.5th training hours. Data collection and analyses were not performed blind to the conditions of the experiment.

#### Psychophysics tasks

There were two psychophysics tasks used for this study, coded using PsychoPy^51^. The tasks consisted of a motion direction discrimination task and an orientation discrimination task. These tasks were selected because they cover two low-level visual characteristics that have been previously studied in the VPL literature. Furthermore, replicating tasks used in prior publications allowed us to use the results from these publications as baseline expectations for our own study. The motion task was based on a VPL study^11^ and provided an estimate of expected optimal transfer (i.e., testing and training are identical). The orientation task was based on a video game learning study^39^ and provided an estimate of expected AVG transfer. Each participant completed the two tasks in a randomized order in each of three tests.

**Fig. 1:**
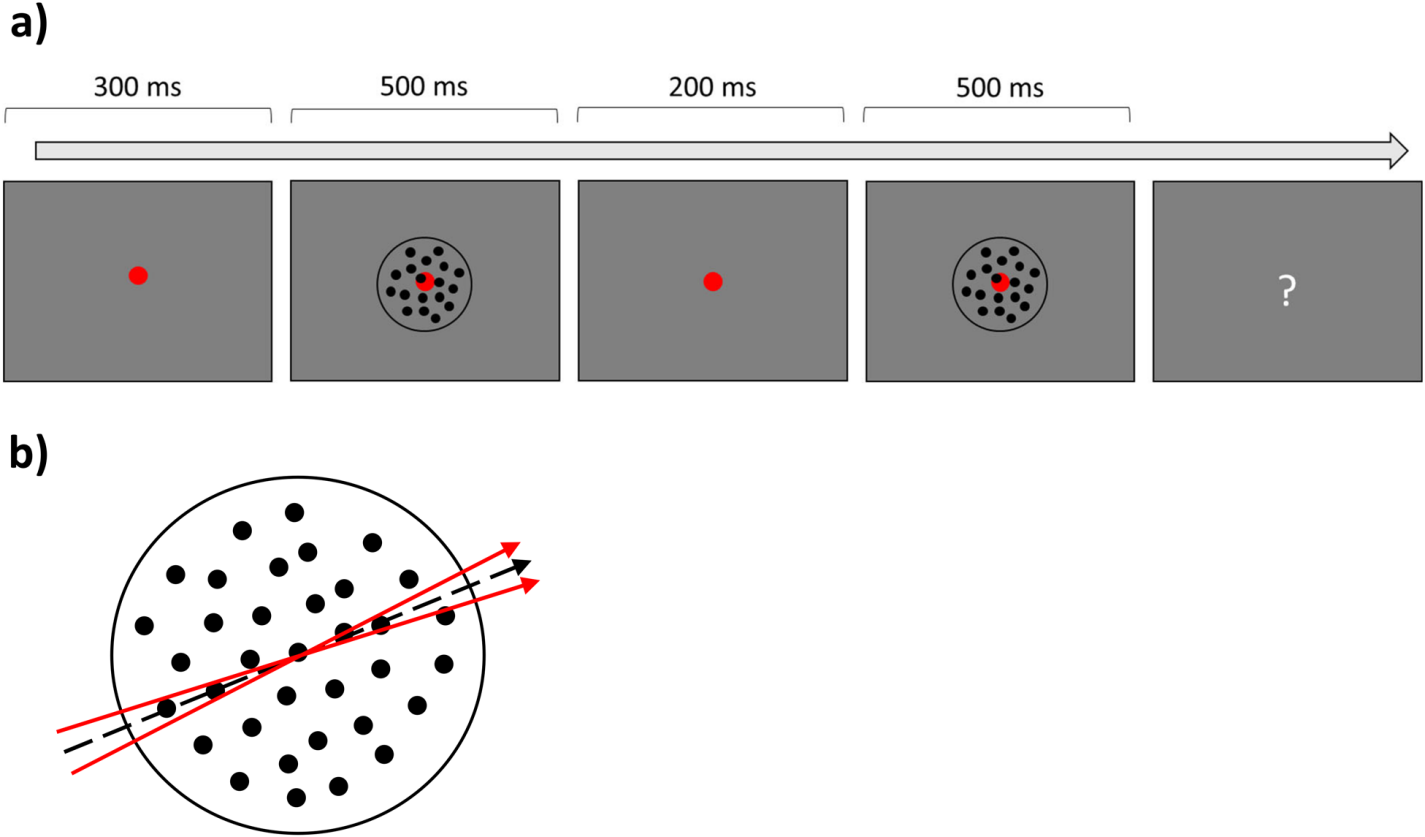
Motion direction discrimination task. (**a**) Example trial procedure for the motion direction discrimination task. (**b**) Each trial’s stimuli contained the same pair of motion directions (solid red arrows): one with a positive offset from the implicit reference angle (75°, dotted black arrow) and one with a negative offset (not to scale in figure). The order of presentation randomly varied from trial to trial.

The motion discrimination task (Fig. 1) was the same as the 2AFC task used in Liang et al. (2015)^11^. Each stimulus was a motion dot stimulus consisting of a borderless circular aperture (8° visual angle in diameter) containing 400 randomly placed dots (0.09˚ in diameter) that were all moving in the same direction at the same speed of 10˚/sec, with a full life time. Each stimulus was presented for 500 ms, with an inter-stimulus interval of 200 ms. The average motion direction of each trial was 75˚ clockwise from vertical, with the two stimuli always separated by + 3° or − 3°. That is, the pair of stimuli always had motion directions of either 73.5° and 76.5°, or 76.5° and 73.5°. There was a central red fixation dot throughout each trial, 0.5˚ in diameter. Participants were asked to indicate which stimulus contained a more clockwise motion direction. Feedback for every trial was provided in the form of a beep if correct.

**Fig. 2:**
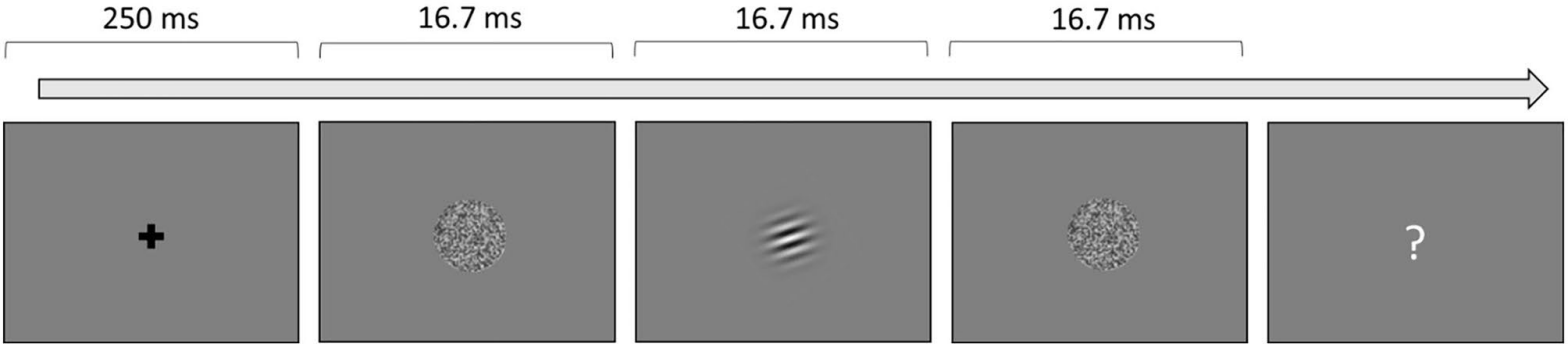
Orientation Task. Example trial procedure for the orientation discrimination task. After stimulus presentation, participants have unlimited time to respond. Once a participant responds, they will receive auditory feedback if they are correct.

The orientation discrimination task (Fig. 2) was exactly the same as the yes–no task reported in Bejjanki et al. (2014)^39^, using Gabors (sine wave grating with a Gaussian filter) viewed through a circular aperture (1.5° visual angle in diameter) as stimuli. All Gabors were identical in spatial frequency (2 cpd), and only differed in orientation. The Gabor was tilted 2° clockwise or counterclockwise from horizontal. Every Gabor was presented and sandwiched in time by external noise. External noise images were the same size and shape as the Gabors, consisting of pixels drawn independently from a Gaussian distribution. Noise energy was increased in task-relevant spatial frequency channels by filtering the external noise images through a band-pass filter with spatial frequencies ranging from one octave below to one octave above the Gabor frequency.

For each trial of the orientation discrimination task, a central fixation cross was presented for 250 ms. A noise image was then presented, followed by the Gabor image, and then followed by another external noise image. Each of the three images was presented for 16.7 ms. Participants responded with a keypress to indicate whether the Gabor was tilted clockwise or counterclockwise from horizontal, and received auditory feedback for a correct answer. There were eight external noise contrast levels (0.0, 2.1, 4.1, 8.3, 12.4, 16.5, 24.8, and 33.0%), presented in an interleaved fashion. Root mean square (RMS) contrast was used. In addition, there were two interleaved staircase procedures used for each level of contrast, one targeting at the threshold of 79.37% correct (3-down-1-up) and the other targeting at the threshold of 70.71% correct (2-down-1-up). Step size was 10% of the current contrast. Contrast threshold was calculated as the mean of all reversals, barring the first four. There were 100 trials for each 3-down-1-up staircase, and 80 for each 2-down-1-up staircase, totaling 1440 trials.

Both tasks were also preceded by practice trials, but with larger angular offsets that were easier to perceive. In the motion task, this angular offset was 20˚ (12 trials at UCLA and 40 at Hebei). In the orientation task, the practice used three offsets that were presented four trials with ± 20˚ offset, four with ± 10˚ offset, and finally four with ± 2˚ offset (at Hebei this practice was repeated once). We note that, although practice was not exactly the same at the two sites, each site had 50–50 distribution between the two training groups. Therefore, no systematic difference should be introduced by this practice difference.

#### Video game

The AVG that participants trained on was *Medal of Honor: Pacific Assault*^52^*.* This game was a commercial first-person shooting (FPS) game, selected because it and other games from the *Medal of Honor* series had been used in prior video game learning studies^32,53,54^. This game, *Medal of Honor*, allowed the player to progress through a series of levels of increasing difficulty, while navigating in a 3D environment and shooting various targets.

The selection of this older video game raised the question of whether it would be relevant to utilize a video game that was no longer comparable to modern video games in terms of gameplay and graphics. However, similar games from the 2000s had commonly been used as training games in most prior video game studies^27,32,55^, including the study whose orientation discrimination task we were attempting to replicate^39^. Given that the claims of AVG transfer from these prior studies had been controversial, it would be important to first verify the results of these previous studies before moving on to more modern games.

#### Training procedure

Participants assigned to the video game training played through the video game starting from the beginning, which was a tutorial level designed to introduce players to the controls of the game. In subsequent sessions, participants proceeded through the levels of the game. For participants assigned to the psychophysics training, they trained on the motion direction discrimination task identical to the one used in the pre- and post-test.

**Fig. 3:**
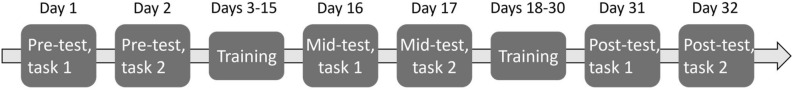
Training and testing procedure. Timeline of training and testing. There were 19.5 h total of training. There were also two 45-min sessions per test, spread over two days. The pre-test occurred before training began, the mid-test after 9.75 h of training, and the post-test after 19.5 h of training.

Participants completed training and testing over the course of 24.5 h. All tests were conducted as two 45-min sessions that took place on two consecutive days, one task per day. The pre-test was conducted before training began, the mid-test after 9.75 h of training, and the post-test after 19.5 h training total. Training lasted for 26 sessions, each 45 min long and on a separate day (Fig. 3).

We opted for a compromised training session length of 45 min for the following reasons. While AVG studies typically used training sessions of at least one hour each, VPL studies relied more commonly on a given number of trials. Furthermore, our observation into the length of VPL training sessions indicated that a session duration of 30–60 min did not encounter performance decrement over time, while longer sessions of 90–150 min did^56^. We therefore chose a training session duration that fell within that shorter range to prevent potential performance decrease in the psychophysics training.

### Sampling plan

#### Participants

Sixty-seven participants were recruited from the University of California, Los Angeles (UCLA) (*n* = 9, seven female, *M*_*age*_ = 20.78 years) and Hebei Normal University, China (*n* = 58, 43 female, *M*_*age*_ = 20.18 years). Participants were first divided into male and female categories, and then within each category randomly assigned to either the video game or motion discrimination training group. At Hebei, this resulted in seven males and 21 females in the video game group (plus two excluded female participants), and eight males and 20 females in the motion training group. In the UCLA cohort, one male and four females were in the video game training group, and one male and three females in the motion training group.

In a subsequent control condition, an additional 29 participants were recruited at Hebei Normal University, but 11 dropped out, leaving 18 remaining (16 female, *M*_*age*_ = 22 years).

Participants in China were recruited using flyers and received monetary compensation. The flyers used to recruit participants indicated criteria for participation, including normal or corrected-to-normal vision and lack of video game experience. Interested participants were asked to complete an online survey before continuing to the experiment itself, in order to verify that they qualified for the experiment and to gather some video game history and demographic information from participants. If a participant was selected, normal or corrected-to-normal vision was confirmed in the laboratory using a Snellen visual acuity chart.

Participants at UCLA were recruited from the lab’s research assistants, and received course credit. These participants were screened by an experimenter for the same criteria for participation as the Chinese participants.

#### Exclusions

Participants were excluded from further analyses if they completed only one test session or fewer.

#### Sample size

Ideally, the sample size could be calculated based on prior effect sizes from the literature. However, for this study, it was difficult to determine which field to draw effect sizes from, since this proposed study combined literature from two fields. In the field of visual psychophysics, studies tended to use a small number of participants, each of which completed a large number of trials. Sample size ranged from three^57^ to 34^58^. Large numbers of trials could also increase reliability of a measurement, which in turn increased the effect size^59^. In our psychophysics training condition when training and testing used the same task, the effect size would be expected to be large. In the literature, similar cases gave rise to a Cohen’s *d* in the range of 1.11^60^ to 2.74^61^.

Nevertheless, there was uncertainty as to how much action video game (AVG) could transfer to a psychophysics task, despite our optimistic hypothesis #1. Based on the effect size for studies targeting perception learning from AVG from a meta-analysis^62^, we chose a conservative estimate, with Cohen’s *d* = 0.227.

To calculate the corresponding sample size n^63^, we focused on the interaction effect between training type and time—that is, will one type of training transfer more than the other for a given test? Accordingly, we calculated the n required for a repeated-measures ANOVA, and found that a total of 64 participants would be required to achieve a power of 0.9, or 32 participants for each of the two training groups (psychophysics and AVG, not including the additional control participants).

### Analysis plan

#### Pre-processing

As indicated previously in the Sampling plan, data would be excluded from participants who completed only one test session or fewer. All data would be included otherwise.

#### Computing thresholds in orientation discrimination

For each participant, we would calculate their threshold for each condition of the orientation discrimination task, as well as an overall mean threshold, at each stage of testing: pre-, mid-, and post-tests. Because the orientation discrimination task used a staircase procedure, we would calculate thresholds by averaging all reversal values except the first four. There would be two thresholds calculated due to the use of two different staircases that corresponded to 79.37% and 70.71% correct in orientation discrimination. We would also calculate the mean percentage improvement (MPI) for each training condition, where MPI = (pre-test threshold−post-test threshold)/pre-test threshold.

#### Computing sensitivity in motion discrimination

For each participant, we would calculate their d’ for the motion discrimination task at each stage of testing. We could calculate the analogous MPI for each motion training condition, which would be (post-test d’−pre-test d’)/pre-test d’.

#### Training scores in video game

Various performance statistics would be compared at each testing point. Training scores would be used to quantify the degree of improvement in video game playing. The hypothesis was that, if AVG indeed could transfer to a psychophysics task, then the amount of game improvement and transfer should be correlated.

### Hypothesis testing

Our basic hypothesis was that, if the video game training group’s improvement in psychophysics from pre- to post-test was greater than the control group’s, then there was transfer. This transfer could be quantified, for motion discrimination, by comparing with the improvement in the motion discrimination training group. This transfer could be also quantified by comparing with the transfer found in Bejjanki et al. (2014). Our second hypothesis regarding possible learning rate difference could be also tested by comparing the improvement from pre- to mid-test with that from mid- to post-test. All these comparisons could be made with ANOVA’s.

#### Equivalence testing

If any of our test results suggested that the AVG and psychophysics training groups were comparable measured in MPI’s, we would conduct an equivalence analysis, which would be conducted using the TOSTER library^63^.

Such an equivalence test also requires a predetermined smallest effect size of interest, or SESOI. We would use *d* = 0.953, for the following reasons^63^. We calculated critical t-values from three studies^9,64,65^ that claimed full transfer in visual perceptual learning. The statistical test used to justify these claims was a one-tailed paired-samples t-test, with a sample size of *n* = 5 or 6. With α = 0.05, we were therefore able to find the critical t-values: 2.132 and 2.015. These t-values were then converted to Cohen’s *d*: 0.823 and 0.953. We selected the larger *d* as our SESOI. A result satisfying the equivalence test would suggest that AVG training fully transferred to the motion discrimination task.

## Results

### Deviations from protocol

Here we list deviations and their rationales below. Analysis details will be placed in relevant result sections for ease of reading.

1. The proposed sample size of 64 was exceeded by three, due to over-recruitment in an attempt to account for possible attrition. Beyond this 67, 11 control and two video game participants, all from Hebei, did not complete the study and were excluded from data analyses. One quit due to cybersickness, six left for academic reasons, and six left for personal reasons. 
2. Our participants were unable to do the orientation discrimination task, at all external noise levels and in all tests. To verify, we fitted psychometric functions to further confirm. We also communicated with three authors of the Bejjanki et al. (2014), which was the basis for this replication task. Author A stated that “there may be an error” in Bejjanki et al. (2014) specifying the stimulus as ±2 deg from the horizontal. Author B checked a PhD thesis, which this experiment was part of. There, ±2 deg was indeed specified. Author C stated that they “used the exact code” from the lab this task was originally started in, that ±2 deg around horizontal had been stated in all presentations, and that the code used could no longer be retrieved. For reference, Lu and Dosher (2004), which Bejjanki et al. (2014)’s method was based on, used ±8 deg with the base orientation of 45 deg.
3. Additional control data (*n* = 18) was collected after the proposed experiment with the AVG and motion discrimination training was completed. This decision was based on the observation from the data collected thus far that the potential confound of test-retest needed to be ruled out. We originally proposed to compare between an AVG training group and a control group to first qualitatively ascertain that there was transfer from video game to psychophysics, before running a motion discrimination training group to quantify the amount of transfer because the amount of learning in a motion discrimination training group had been quantified already in the literature. This was declined. We could not simultaneously run the three training groups suggested: action video game, non-action video game, and motion discrimination, because it would have required 96 trainees, each going through full training and testing. In subsequent data analysis, therefore, we would conducted a 3x3 ANOVA.
4. While psychophysics studies typically focus on accuracy or discrimination sensitivity, a prior video game study found that a motion task similar to ours could receive transfer from AVG in the form of faster reaction time. We hence conducted an additional analysis on reaction time data in the motion task to further verify lack of transfer from AVG training.
5. We originally proposed to analyze the data using, e.g., learning rate and MPI (mean percent improvement). However, after data were collected, we realized that some of the hypotheses could be tested better and more directly, without using these measures. Therefore, in the data analyses, we used more straightforward methods when possible.

### Orientation discrimination

It turned out that most trainees’ staircases did not converge, but oscillated near the maximal stimulus contrast of 1.0, suggesting that the task was too difficult. Consequently, rather than computing an average from the reversals, as was done in Bejjanki et al. (2014), we used logistic regression to fit a psychometric function for each participant at each external noise level: $y=1-0.5*{e}^{{{-(x/a)}{b}^{2}}}$, where x = stimulus contrast and a > 0. The binary behavioral response (0 or 1) is fitted by the continuous y.

**Fig. 4:**
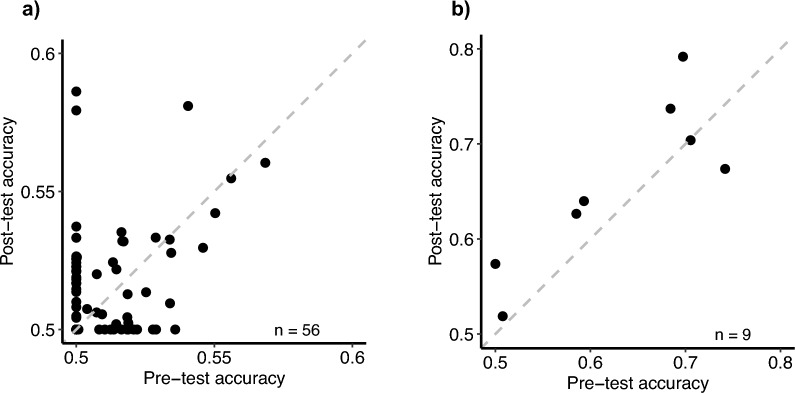
Pre-test vs. post-test accuracy on the orientation discrimination task. Each participant’s pre-test and post-test data from the orientation discrimination task was fit to a psychometric function, which was used to calculate predicted accuracy when the stimulus contrast = 1: (**a**) from the Chinese participants, (**b**) from the UCLA participants.

We then estimated the accuracy from each curve fitting when the stimulus contrast = 1.0 (Fig. 4). None of the accuracies exceeded 55% from our Hebei participants (*n* = 52). Of the nine UCLA participants, seven exceeded 55%. We hence compared these accuracies pre- and post-training, for each of the eight external noise levels. For each participant, we counted the number of external noise conditions where this accuracy increased. If more than half of the conditions showed increases, this participant would be labeled as a learner. Five of the seven participants were learners. However, this result could not reject the null hypothesis that the probability for a participant to improve was 0.5 (*p* = 0.16). We concluded therefore that, irrespective of their training condition, the participants did not improve their orientation discrimination. This means that we could not replicate the main finding in Bejjanki et al. (2014), although this could simply be due to the fact that the task was too difficult for our participants.

### Motion discrimination

**Fig. 5:**
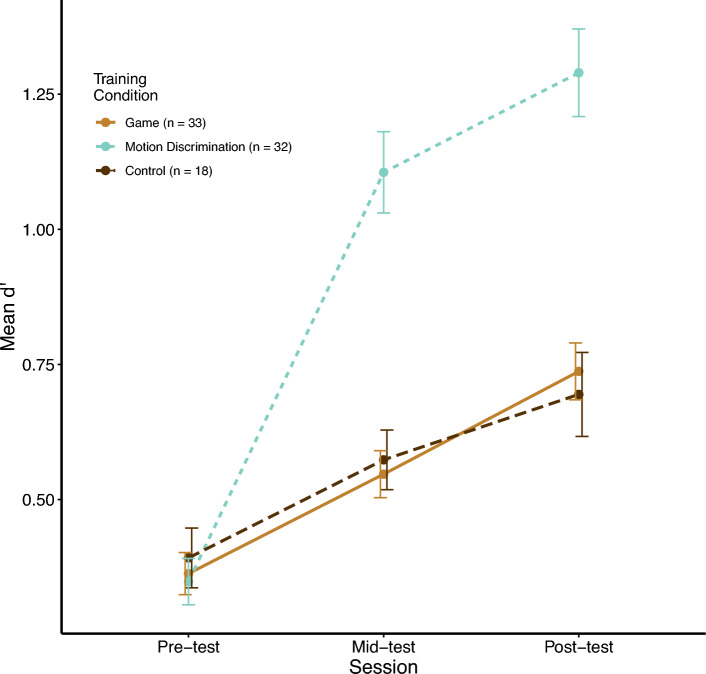
Mean sensitivity over the course of training. Participants trained on an FPS video game or a motion discrimination task for 20 h, or were in a no-contact control group. They were tested on a motion discrimination task before, midway, and after training. Mean group d’ (sensitivity) on motion discrimination is plotted at each test point. Error bars represent ± 1 standard error, as error bars in subsequent figures. No difference was found between the control and video game training group, but the motion discrimination training group improved significantly more than the other groups.

Discrimination sensitivity (d’) was calculated from 720 trials at each test point (pre-, mid-, and post-test) for each participant from all three groups. A type III mixed-subjects ANOVA was conducted on these data, with training condition (video game, motion, control) as the between-subjects factor and test session as the within-subjects factor. All effects were statistically significant: test (*F*(1.49, 113.08) = 138.80, *p* < 0.001); training (*F*(2, 76) = 14.30, *p* < 0.001); and interaction (*F*(2.98, 113.08) = 25.24, *p* < 0.001) (Fig. 5).

Follow-up one-way ANOVAs and t-tests were conducted, particularly in comparisons between the video game training and control groups. At pre-test, all three training groups (control: *M* = 0.39, *SD* = 0.23; video game: *M* = 0.36, *SD* = 0.22; motion: *M* = 0.35, *SD* = 0.24) performed comparably (*F*(2, 80) = 0.20, *p* = 0.82). At mid-test, a significant one-way ANOVA (*F*(2, 80) = 27.81, *p* < 0.001) prompted further t-tests. No significant difference was found between the control (*M* = 0.57, *SD* = 0.23) and video game training group (*M* = 0.55, *SD* = 0.25) (*t*(37.1) = 0.38, *p* = 0.71). However, the motion group (*M* = 1.11, *SD* = 0.43) showed greater sensitivity than the control group (*t*(48) = 5.70, *p* < 0.001, Cohen’s *d* = 1.55) and the video game training group (*t*(49.9) = 6.43, *p* < 0.001, Cohen’s *d* = 1.60). Finally, at post-test a one-way ANOVA was again significant (*F*(2, 80) = 22.51, *p* < 0.001). Importantly, the pattern from mid-test comparisons persisted: between controls (*M* = 0.69, *SD* = 0.33) and gamers (*M* = 0.74, *SD* = 0.30) (*t*(33.2) = -0.64, *p* = 0.53), between motion (*M* = 1.29, *SD* = 0.46) and controls (*t*(44.4) = 4.95, *p* < 0.001, Cohen’s *d* = 1.49), and between motion and gamers (*t*(53.4) = 5.72, *p* < 0.001, Cohen’s *d* = 1.42). These results suggested no evidence that video game training transferred to motion discrimination.

### Correlation between video game training and motion discrimination tests

**Fig. 6:**
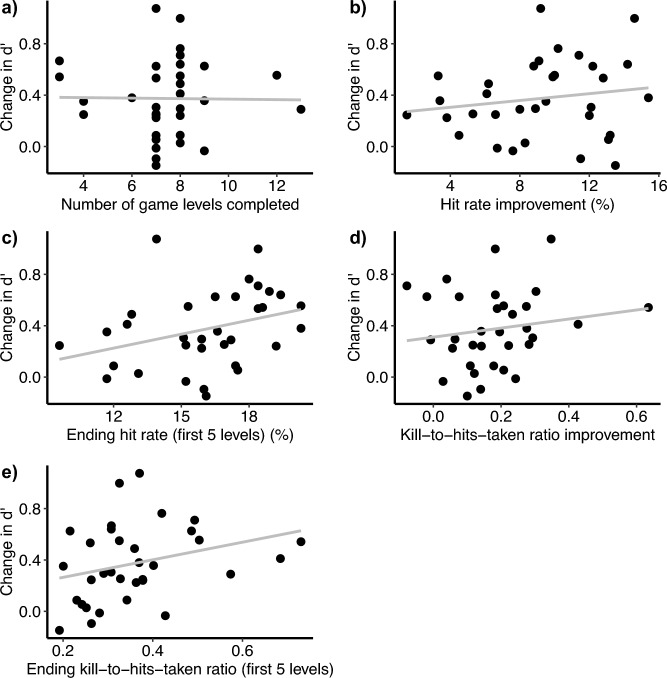
Correlations between gaming improvement measures and d’ increase. All correlations are between a training measure and individual improvement in d’ (sensitivity). Training measures depicted are (**a**) Number of game levels completed; (**b**) Improvement in hit rate (calculated from the ratio of the number of hits landed on enemy targets to the number of shots fired) over the course of playing the first five levels; (**c**) Ending hit rate after the first five levels; (**d**) Improvement in ratio of kills to hits taken (the number of enemies successfully killed versus the number of times the player was hit by enemy fire) over the first five levels; and (**e**) Ending ratio of kills to hits taken after the first five levels. All correlations were small and not statistically significant.

As an additional check on any possible transfer from video game training to motion discrimination, we calculated a number of indexes for video game improvements, and then correlated each with improvement in d’ in motion discrimination per video game trainee, from pre- to post-test (Fig. 6).

- Number of game levels completed. A one-sample t-test confirmed that the video game trainees reached levels beyond the first (*t*(32) = 21.1, *p* < 0.001). The correlation coefficient between game level and improvement in motion *d’* was -0.013, however.
- Improvement in hit rate within the first five levels, which constituted the first stage of the game. We only used these five levels because not all participants progressed beyond them. This hit rate improvement was quantified as a participant’s hit rate after completing the first two levels (in order to avoid unreasonably high hit rate after the introductory level), subtracted from the participant’s hit rate at the end of training or the completion of these five levels, whichever came first. A paired-sample t-test confirmed that the participants significantly improved this hit rate (*t*(32) = 14.5, *p* < 0.001). The relevant correlation coefficient, however, was 0.16 and not statistically significant (*t*(31) = 0.91, *p* = 0.37).
- Hit rate upon completion of training or the first five levels of the game, whichever came first. The corresponding correlation coefficient was 0.29, but was still not significant (*t*(31) = 1.94, *p* = 0.062).
- Modified kill-death ratio (KDR). KDR was a common measure of performance in first-person shooter video games, and had been used in prior studies^66,67^. Since our game did not record player deaths, we substituted the death number by the number of hits taken by the player. We looked at the improvement over the first five levels, or over the course of training, whichever was shorter. A t-test confirmed that participants significantly improved (*t*(32) = 7.45, *p* < 0.001). The correlation was 0.16, not significant (*t*(31) = 0.92, *p* = 0.37).
- The modified KDR at the end of training or the first five levels of the game, rather than its improvement as in d). The correlation was 0.29, again not significant (*t*(31) = 1.70, *p* = 0.099).

### Exploratory analyses

#### Reaction time

Green et al. (2010)^40^ reported that active video game training reduced participants’ reaction time (RT), whereas control participants’ RT was not reduced, even though no accuracy difference was found between them (see also^53^). We accordingly looked into our participants’ RTs, both their means and medians.

A mixed-subjects ANOVA on mean RT data from the three groups revealed only a significant main effect for test time (*F*(1.76, 141) = 29.94, *p* < 0.001). The main effect for training group and the interaction were not significant (*F*(2, 80) = 1.31, *p* = 0.28; *F*(3.53, 141) = 2.13, *p* = 0.089).

**Fig. 7:**
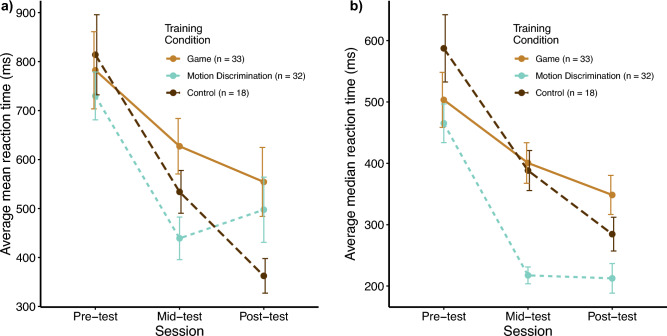
Reaction times over the course of training. a) Mean reaction time (RT) for each training group and the control group at each testing point, calculated from individual mean RT data at each time point. All groups show significant decrease in reaction time, with the control group showing significantly decreased reaction time at post-test compared to the other two groups. b) Mean RT data, calculated from individual median RT data at each time point. All groups decreased reaction times significantly over time, but the motion discrimination training group experienced the largest overall decrease. The video game training group and control group did not have statistically significantly different reaction times at any time point.

A similar ANOVA on median RTs found main effects for test (*F*(1.4, 111.85) = 78.92, *p* < 0.001) and for group (*F*(2, 80) = 6.56, *p* < 0.01). A significant interaction was also found (*F*(2.8, 111.85) = 4.22, *p* < 0.01). As seen in Fig. 7, unlike in Green et al. (2010), all training groups improved in median RTs over time, but the psychophysics training group consistently had faster improvement. It is unclear why there is such discrepancy, except that we used 3° direction discrimination with 100% motion coherence, whereas Green et al. (2010) used 180° direction discrimination with a range of motion coherence. We also note that no RT reduction was found in van Ravenzwaaij et al. (2014)^28^ and Pavan et al. (2016)^68^, although the latter found discrimination sensitivity transfer in parafoveal vision.

## Discussion

Prior research suggested that playing AVGs benefited a wide variety of visual tasks^32,62,69^, implying that playing AVGs transferred broadly and effectively to vision. Therefore, we investigated how much playing an AVG would transfer to lower-level visual tasks such as orientation discrimination and motion direction discrimination. Participants with no significant AVG experience were recruited and trained with either AVG or motion discrimination. Motion and orientation discrimination was assessed before, in the middle of, and after training. We hypothesized that, based on prior literature, participants trained on AVG would transfer their learning to visual tasks. We asked how effective transfer would be from AVG as compared to from motion discrimination training itself, to motion and orientation discrimination.

Although the AVG group improved their motion discrimination from pre- to post-test, the control group improved comparably. This indicated that AVG training did not transfer to motion discrimination and was unnecessary for such improvement, because the controls had no training in the same duration from pre- to post-test. This result further indicated that a passive video game training was no better a control than our no-contact control.

We also analyzed reaction time data, since Green et al.^40^ had found that AVG training reduced reaction times in their motion discrimination. Our analysis was consistent with the d’ analysis above. Namely, that reaction times were reduced with statistical significance for all participants, but more so for the motion training group. The video game training group and the controls showed comparable reduction, indicating that such reduction was due to test-retest and not to transfer from AVG training to motion discrimination.

Correlational analyses of the AVG training group further supported such conclusion of no transfer. Among all the metrics that we could think of that measured AVG improvement, none correlated with the same participant’s motion discrimination improvement. Note also that these metrics above all indicated AVG improved with statistical significance.

Our orientation discrimination task was intended to replicate the same task in Bejjanki et al.^39^, which was one of only a few studies demonstrating transfer from AVG to a low-level psychophysics task. Unfortunately, this task proved to be too difficult for our participants. Experimenter error was the cause for not detecting this difficulty earlier, in part because the ceiling of stimulus contrast at 1.0 made the staircases often appear to be converging. Aside from the possibility that ± 2° in Bejjanki et al.^39^ was a typo, our best explanation for our participants’ chance performance is that we did not pre-train them enough. Prior to our study, we carefully studied Lu and Dosher (2004), on which Bejjanki et al.^39^ was based. However, we did not find specifications regarding pre-test practice.

Our hypothesis for potential broad transfer to a variety of laboratory cognitive and perceptual tasks from video game learning was varied training—that exposure to a diverse range of stimuli and demands would facilitate general rule learning that subsequently enables transfer to a wide range of stimuli and tasks^5,23,70^. However, our results did not support such a hypothesis. What might be the explanation?

1. One possibility is that 20 h of video game training is insufficient. Although one meta-analysis found that 20 h was within the effective range, this was at the lower bound^44^. It could be that, for transfer to happen to low-level psychophysics tasks such as motion discrimination, longer training was necessary. Indeed, gamers can easily spend 15 h or more per week playing, which would within two weeks surpass the amount of our participants’ training. This possibility is consistent with results from cross-sectional studies that directly compared video game and non-video game players, and with training studies that investigated effects of video game training in the laboratory^44,62^. That said, we note that multiple studies reported visual skill transfer from AVG with only about 10 h of training, although such skills were not necessarily as low-level as motion discrimination^32,67,71^.
2. The second possibility is that AVG training cannot transfer to low-level VPL, no matter how long training takes. After all, the varied learning remains a theory that may or may not apply to visual perceptual or other kinds of learning^72^. It remains possible that motion perceptual learning, as studied currently, is primarily template learning and untransferable from higher-level, rule-based learning. Similar lack of transfer had been found in prior studies that compared AVG training with various control conditions, although none of the prior research has tested motion direction discrimination^27,73,74^.

Finally, the current study was set out to quantify the relative amount of transfer from AVG training as compared to training with the same visual perceptual task, under the assumption that AVG training could transfer. Such an assumption was made as a result of numerous studies claiming transfer from AVG training, particularly to psychophysics tasks^38,39^. Nevertheless, such an assumption remains an assumption until verified empirically.

In our case, due to the initial ambiguous results from the AVG training group that improved in motion discrimination, controls were added to verify whether such improvement was simply due to test-retest, unrelated to any transfer. Here we address two objections against this use of the control.

1. One objection was that such controls were below standards in AVG literature, in demand characteristics, and placebo/expectation effects. This objection suggested that no-contact control would be insufficient to account for everything beyond AVG training that would contribute to the improvement. However, our results indicated that such controls already matched the full effect (or the lack thereof) the AVG training. Therefore, any possible effects due to demand characteristics and placebo/expectation must be negligible, at least in our case.
2. Another objection was that, should the controls be selected after AVG and motion training groups, rather than simultaneously, random selection would be compromised. We had found^10^, however, whether a group was selected simultaneously with another group, or afterwards, made little difference so long as everything else was unchanged. It appeared that, at least in this context, participants in a fall semester performed similarly to their counterparts in a spring semester.

There are studies that have explored video games therapeutically, in treating amblyopia^37,54,75^ and dyslexia^76,77^. These studies generally found promising results. Thus, video games may have the potential to affect vision, but the mechanism by which they do so might not affect low-level vision. While our results suggested video games were limited in the extent to which they transferred, these results also provide evidence to narrow down the mechanism by which video games are able to affect visual learning.

## Supplementary Information

Supplementary Information.

## Data Availability

All data and materials are available on the Open Science Framework at https://osf.io/zdv9c/?view_only=5b3b0c161dad448d9d1d8b14ce91ab11.

## Code availability

All analysis code is available on the Open Science Framework at https://osf.io/zdv9c/?view_only=5b3b0c161dad448d9d1d8b14ce91ab11.
