# Supplementary material for: Comparing conventional and action video game training in visual perceptual learning
Source: Sci Rep. 2024 Nov 13;14:27864. doi: 10.1038/s41598-024-71987-y (PMC11561280; doi:10.1038/s41598-024-71987-y)
Supplement: Supplementary file 1 — Supplementary Information. [file 41598_2024_71987_MOESM1_ESM.docx]

**Comparing conventional and action video game training in visual perceptual learning**

Maggie S. Yeh^1^*, Jinfeng Huang^2^, and Zili Liu^1^

^1^ Department of Psychology, University of California Los Angeles, Los Angeles, CA, USA

^2^ Hebei Normal University, Shijiazhuang, China

* Corresponding author: Maggie S. Yeh ([maggieyeh@g.ucla.edu](mailto:maggieyeh@g.ucla.edu))

**Supplementary Information**

**Pre-experiment survey**

**Section 1: Demographic Information**

Participant ID: ________________

Gender: _______________

Age: ______

Are you prone to motion sickness? Y/N

Do you have normal or corrected-to-normal vision? Y/N

Do you have extensive prior experience with psychophysics tasks? Y/N

**Section 2: Video game experience (recent)**

This section is asking about your video game experience from the past 12 months.

Have you played video games at all in the past 12 months? Y/N

(If no, participants will be directed to the next section)

Of the video games you have played in the past 12 months, did you play any games on a console combined with a TV screen? (e.g. Playstation, Xbox) Y/N

If yes, how often did you play game(s) on consoles per week? ____________

How long, on average, was each session of playing games? ____________

Please list up to 5 games that you played most often on a console. _____________

Of the video games that you have played in the past 12 months, did you play any games on a handheld console? (e.g. Nintendo DS) Y/N

If yes, how often did you play game(s) on a handheld console per week? _____________

How long, on average, was each session of playing games? ______________

Please list up to 5 games that you played most often on a handheld console. _____________

Of the video games that you have played in the past 12 months, did you play any games on a laptop or personal computer? Y/N

If yes, how often did you play game(s) on a laptop/PC per week? _____________

How long, on average, was each session of playing games? ______________

Please list up to 5 games that you played most often on a laptop/PC. _____________

Of the video games that you have played in the past 12 months, did you play any games on a smart phone or tablet? Y/N

If yes, how often did you play game(s) on a phone/tablet per week? _____________

How long, on average, was each session of playing games? ______________

Please list up to 5 games that you played most often on a phone/tablet. _____________

Of the video games that you have played in the past 12 months, did you play any games on a VR device (e.g. Oculus Rift) Y/N

If yes, how often did you play game(s) on a VR device per week? _____________

How long, on average, was each session of playing games? ______________

Please list up to 5 games that you played most often on a VR device. _____________

**Section 3: Video game experience (overall)**

Do you have significant experience playing video games from before the past 12 months? (Significant experience can be considered as over 20 hours spent playing games) Y/N

If yes, when did you last play video games? ___________

Roughly how many hours per week did you play games? _________

Please list up to 5 games that you played most often. _____________
